# Why so little progress in therapeutic thrombolysis? The current state of the art and prospects for improvement

**DOI:** 10.1007/s11239-015-1217-3

**Published:** 2015-04-18

**Authors:** Victor Gurewich

**Affiliations:** Vascular Research Laboratory, Mt Auburn Hospital, Harvard Medical School, Cambridge, MA USA

## Introduction

Thrombosis is a major contributor to disability and mortality worldwide and is estimated to be the cause of one in four deaths [1]. It is the pathology responsible for triggering acute myocardial infarction (AMI) and ischemic stroke, and is the primary cause of venous thromboembolism. The pathogenesis has been well-studied starting with Virchow more than 150 years ago [2], and its prevention by anti-thrombotic drugs has made much progress, culminating most recently in the approval of new oral anticoagulants which are specific inhibitors of thrombin or factor Xa.

By contrast, treatment by thrombolysis, the only pharmacological means available, has remained at a near standstill since Genentech first applied for FDA approval of tissue plasminogen activator (tPA) in 1983. A replacement for streptokinase (SK) had been long-sought, but after early successes in AMI, tPA use has since been on the decline.

Instead, percutaneous coronary intervention (PCI) has become the treatment of choice in AMI, despite its technical, time-consuming, and costly demands. Pre-treatment with tPA to hasten coronary reperfusion, so-called facilitated PCI, was found to increase PCI complications including intracranial hemorrhage (ICH) making the two incompatible [3]. In ischemic stroke, tPA has been estimated to be used in only 2–5 % of patients over-all [4], and in pulmonary embolism, bleeding and stroke complications limited its use to the minority of patients with hemodynamic decompensation [5].

The recent trend in stroke has also begun to favor the use of endovascular devices with three major trials in ischemic stroke published in the first 2 months of 2015 in the NEJM [6–8]. In this same period, a clinical trial of catheter-directed ultrasound was also published which showed a significant acceleration of the thrombolysis rate by this method [9].

These device reperfusion techniques require bringing the patient to a qualified facility which inevitably consumes precious time for conditions in which “minutes are myocardium or neurons.” An optimal treatment for these time-sensitive conditions can only be one that can be brought to the patient and is easily and safely administered. The resort to alternative methods is a reflection on the inadequacy of current thrombolysis, a problem that was foretold by the results with tPA in major clinical trials.

## A brief review of thrombolysis with tPA

TPA was the first fibrin-specific thrombolytic [10] and replaced SK, which was non-specific, causing extensive hematological side effects, and which was an indirect plasminogen activator. By contrast, tPA is a direct activator and one that is strongly promoted by fibrin to which it has a high affinity [11]. In the same year, this property was shown to be sufficient to purify tPA directly from human plasma in a single step [12].

In 1983, tPA was successfully produced by recombinant technology by Genentech [13], and AMI was selected as the first disease target. It was anticipated that because of its fibrin-specific mode of action, tPA would prove far more effective than SK, as also suggested by preliminary trials [14]. However, their difference turned out to be so marginal that it required an unprecedented total of 94,720 patients to arrive at a statistically significant *p* value in comparative clinical trials.

### Summary of the three mega trials of tPA versus SK in AMI

1. GISSI-2 [15]
  In a multicenter trial, 12,490 patients were randomized to either single-chain tPA (100 mg infused iv over 3 h) or a standard dose of SK. No difference in the 30 days death rate was found, which was 9 % for tPA and 8.6 % for SK. The incidence of congestive heart failure, the second endpoint, was also not different. The complication rate of stroke (1.1 and 0.9 % for tPA and SK respectively) and major bleeding (0.5 and 1 %) were also similar.
2. ISIS-3 [16]
  A total of 41,229 patients were randomized to standard doses of double chain tPA (Duteplase), SK, or anisoylated SK (APSAC). All patients received aspirin and half of each group received sc heparin starting at 4 h post thrombolysis. The outcome of the study was similar to that of the previous one with a 35 days mortality of 10.3 % for tPA, 10.6 % for SK, and 10.5 % for APSAC. A significant excess of total stroke with tPA was, however, found, being 1.39 % for tPA and 1.04 % for SK (p < 0.01).
  Once again, no explanation for the surprising lack of clinical benefit from tPA over SK was offered.
3. The GUSTO study [17]
  The final multinational study was with 42,021 patients divided into four groups, two with SK plus either sc or iv heparin and two with tPA and iv heparin, in one of which the tPA was administered by a new accelerated regimen. Only in the group given tPA by the accelerated regimen, was there a statistically significant difference found. The 30 day mortality in this group was 6.3 % with tPA and 7.2 % for SK (p < 0.001). The ICH incidence again was significantly (p = 0.03) higher with tPA than SK, as was the incidence of “moderate or worse” bleeding (p = 0.02).

The finding of a significant difference in the result from this trial was met with some skepticism. In a commentary by three prominent experts, it was concluded that there was “at most, a small absolute difference… in both lives saved and major complications between tPA and SK” [18]. The GUSTO trial results also underwent a Bayesian analysis which found them to be inconclusive and that “the clinical superiority of tPA over SK remains uncertain” [19].

Nevertheless, tPA was given approval for AMI treatment after the GUSTO trial and tPA or one of its longer half-life derivatives, has remained the thrombolytic of choice ever since becoming essentially synonymous with thrombolysis. In 1996, tPA was also approved for the treatment of ischemic stroke.

The unprecedented number of patients that were required to gain approval for a new thrombolytic had its adverse consequences. The experience not only discouraged further investment in thrombolysis by industry, but it also dampened scientific interest. It was believed that the trials showed that all activators must be more or less comparable and that the difference between a fibrin-specific and a non-specific activator was less important than had been believed. It was as if the limits of what was possible with thrombolysis had been reached.

In retrospect, a crucial alternative interpretation was missed. This was that the very nature of tPA’s fibrin-specific mode of action limited its fibrinolytic effect and conversely, that it was the non-specific mode of action of SK that gave it an unrestricted plasminogen activating effect which gave it a comparable efficacy. The activators’ net effect was similar but for very different reasons. The more limited fibrinolytic effect of tPA had been previously implied by the finding that its fibrin-dependent plasminogen activation was different and complementary to that of the other natural plasminogen activator prourokinase (proUK) [20].

## The biological pathway of fibrinolysis and its lessons

Since all therapeutic thrombolysis utilizes the endogenous plasminogen-plasmin pathway, endogenous fibrinolysis provides a useful guide to its function and utilization. For hemostasis, fibrin must be sufficiently stiff to serve as a meshwork to prevent bleeding but at the same time it must be capable of being lysed when vital blood flow is threatened by the same meshwork. Plasmin is responsible for fibrinolysis and does so by cleaving the polymer into two major soluble fibrin degradation products [21], a remarkably efficient solution to the problem. Plasminogen, the proenzyme precursor of plasmin, is available in relatively high abundance in plasma (~2 µM), more than sufficient for its therapeutic utilization.

Plasmin is relatively non-specific and hydrolyzes other substrates including three clotting factors, and can induce a hemorrhagic diathesis. To make it more fibrin-specific, two additional factors have evolved. First plasmin(ogen) targets fibrin by binding directly to certain lysine binding sites [22]. This both targets and promotes its fibrinolytic activity. Unbound plasmin, by contrast, induces little fibrinolysis, as evidenced by microplasmin which has no fibrin binding domains and induces little fibrin degradation but retains a full fibrinogenolytic effect [23]. Second, the two endogenous activators of plasminogen, tPA and proUK, have high specificities for fibrin-bound plasminogen over that which is not bound. As a result, the biological system is highly fibrin specific and proteolysis is additionally limited to the fibrin clot environment by certain inhibitors in the ambient plasma. Importantly, the endogenous system utilizes both activators for fibrinolysis rather than just one as has been the custom in therapeutic thrombolysis.

### The sequential and complementary mechanisms of fibrin-dependent plasminogen activation by tPA and proUK

When intravascular thrombosis occurs, tPA stored in the vessel wall at that site is released, binds to the thrombus, and initiates fibrin degradation. This is mediated by tPA’s high fibrin affinity for a specific site on the D-domain of intact fibrin [11, 24] that is adjacent to fibrin-bound plasminogen. This ternary complex promotes plasminogen activation by tPA more than 1000-fold [25] and efficiently initiating degradation of the fibrin surface. This creates new plasminogen binding sites [26], and plasminogen bound to these new sites is activated preferentially by proUK and its activated form urokinase (UK). Specifically, proUK has a high substrate affinity for plasminogen bound to a triple carboxyterminal lysine high affinity site on the fibrin E-domain of degraded fibrin [27]. Plasminogen at this site undergoes a conformational change which promotes proUK’s intrinsic activity more than 250-fold, giving it an activity equivalent to that of UK [28]. A similar promotion of proUK’s intrinsic activity by plasminogen bound to solid-phase fibrin after its degradation was also shown in another study [29].

This activation of plasminogen by proUK is accompanied by reciprocal activation of proUK by plasmin [30] that is associated with a hypercatalytic transitional state between proUK and UK [31]. Thereafter, UK activates the remaining plasminogen on degraded fibrin. The activation of proUK during lysis adds to its fibrinolytic effect as illustrated by a study with an inactivatable, plasmin-resistant mutant proUK which was 100-fold less effective in clot lysis [32]. ProUK/UK, therefore, has a dual plasminogen-activating function in fibrinolysis. By contrast, tPA undergoes no functional change since its one and two-chain forms have similar fibrinolytic properties [33]. This relatively more limited effect of tPA was observed in an earlier study in which the rates of fibrin-specific clot lysis (<10 % fibrinogenolysis) in a plasma milieu by tPA and proUK were compared. The lysis rate by proUK was consistently twice that of tPA [34], a finding which indicated that proUK activated twice as many fibrin-bound plasminogens.

These findings suggest that there are a total of three different plasminogen binding sites available on fibrin, one on intact fibrin activated by tPA and two additional ones on degraded fibrin activated by proUK and UK, consistent with the 2:1 ratio of their lysis rates. This same number of plasminogens was documented in a previous study in which the molar amounts of plasminogen on intact and degraded fibrin were measured. There were 23.4 nmol/l of plasminogen found on intact fibrin and 61.9 nmol/l on degraded fibrin for a total of three [35].

The fibrinolytic activities of tPA and proUK are, therefore, promoted by different fibrin domains which are the D and E domains respectively. This was also shown in a kinetic study with soluble fibrin fragments D and E in which plasminogen activation was measured. Plasminogen activation by tPA was promoted only by fragment D, whereas that by proUK was promoted only by fibrin fragment E [36], making the activators complementary in their fibrin-dependent plasminogen activating effects.

### Clinical implications

These fibrinolytic differences also affect the etiology of the hemorrhagic side effects of tPA and proUK. Hemostatic fibrin, being protected from fibrinolytic degradation, remains intact and consequently is much more vulnerable to lysis by tPA than by proUK. This is not a problem physiologically because tPA normally circulates as an inactive complex with its inhibitor, plasminogen activator inhibitor-1 (PAI-1), but PAI-1 is at far too low a concentration for therapeutic amounts of tPA, leaving hemostatic fibrin unprotected. This explains the unexpected finding of a significantly higher incidence of ICH by tPA than by non-specific SK in the mega trials [16, 17]. The lysis of hemostatic fibrin by tPA was previously cited as the principle cause [37, 38]. Therefore, bleeding is a direct consequence of tPA’s mechanism of action of targeting intact fibrin to initiate lysis, explaining also why its therapeutic doses need to be limited.

This tPA effect was studied experimentally in a dog model in which blood loss from standardized hemostatically sealed fresh injury sites was measured. Blood loss by tPA was tenfold higher than that by proUK (mean 40 vs 4 ml, p = 0.026) at equivalent fibrinolytic doses [39], a finding consistent with the differences in their mechanisms of fibrin-dependent plasminogen activation.

The etiology of proUK’s bleeding side effects is quite different. ProUK is vulnerable to conversion to UK at therapeutic concentrations in plasma due to plasmin generation at these concentrations. UK compounds this problem by generating much more plasmin inducing a hemorrhagic diathesis with degradation of clotting factors like fibrinogen, as seen in the proUK clinical trials [40]. It was for this reason that, proUK was denied marketing approval, in Europe. To address this problem, a mutant form of proUK was developed (see below).

## Synergistic fibrinolysis by tPA and proUK in vitro and in vivo

When tPA and proUK were used together versus either activator alone, a synergistic effect was observed in clot lysis experiments in a plasma milieu [41] which was attributed to their complementary modes of action [20]. Nevertheless, the synergistic effect of tPA and proUK has continued to remain controversial since it was contested by other investigators [42].

The effect of a sequential combination was also evaluated clinically in a multicenter study of patients with AMI. In the PATENT trial, 101 patients were treated with a small bolus of tPA followed by a modest infusion of proUK [43]. The first 10 patients were given a 10 mg bolus which turned out to be excessive. The remainder were given a 5 mg bolus of tPA (5 % of the standard dose) followed by a proUK infusion of 40 mg/h (50 % of the standard rate) for 90 min. A TIMI 2–3 coronary patency rate of 77 % and a TIMI 3 patency rate of 60 % were obtained, with no reocclusions or strokes and a mortality of 1 %. The patency rate obtained was comparable to that reported for the best of the four groups in the GUSTO trial [44], in which the mortality was 6.3 % with 1.5 % strokes [17].

Despite the success of the PATENT trial, no follow-up study was possible since Farmitalia, the sponsor, was sold soon after and proUK development ceased, and was discontinued by Gruenenthal a little later after their submission for marketing approval of proUK was denied due to the UK bleeding complications.

## The clinical experience with proUK in brief

ProUK, the native, precursor of UK, was first isolated from human urine and identified to be a single-chain proenzyme in 1981 [12, 45]. It was shown to have a fibrin-specific mode of action, in contrast to UK, despite having no fibrin clot affinity [46]. After several small clinical studies with proUK for different indications, phase III clinical trials in AMI were done with recombinant proUK from *E. coli* by Gruenenthal [47, 48]. An efficacy comparable to that of other activators was found but with a lower rate (0–5 %) of rethrombosis and no hematological evidence of thrombin generation [49], in contrast to tPA. However, at the therapeutic concentrations of proUK used, its relatively high intrinsic activity [50] induced systemic plasmin generation which converted proUK to UK, inducing extensive (80–90 %) fibrinogenolysis [40]. As noted above, due to this problem, marketing authorization for proUK was denied by the European Medicines Agency in 1998, “on the basis of the higher incidence of hemorrhagic strokes,” and proUK development was terminated.

Since proUK had unique properties and therapeutic potential, especially in combination with tPA, it was decided to address the problem of its vulnerability to UK conversion by recombinant technology.

## Mutant proUK (HisproUK)

Structure–function studies revealed a flexible loop in the catalytic domain of proUK with a charged residue (Lys 300) at its tip which was shown to be responsible for proUK’s relatively high intrinsic activity [51]. By reducing the charge at this position, the intrinsic activity could be modulated [52]. A single site mutant (Lys300 → His) was eventually selected which had a fivefold lower intrinsic activity but a preserved two-chain enzymatic activity and other properties of proUK were also preserved. The HisproUK mutant was stable in plasma at a fivefold higher concentration than native proUK.

In a dog model, HisproUK induced effective lysis of venous thromboemboli which was associated with surprisingly little bleeding from hemostatic sites [53]. This phenomenon was tested in a second dog study against an arterial thrombus. HisproUK induced a vessel patency rate equivalent to that of tPA, but with tenfold less blood loss from fresh hemostatic sites than tPA [39], as also described above. The low bleeding rate was related to a novel inhibitor complex between HisUK and plasma C1 esterase inhibitor (C1INH) in the plasma of these dogs, which inhibited HisUK mediated side effects.

### The C1INH effect

The same HisUK:C1INH inhibitor complex was found in human plasma after incubation with HisUK. In clot lysis studies in a plasma milieu, C1INH inhibited fibrinogenolysis by HisproUK without interfering with fibrinolysis [54]. Since C1INH is available as a pharmacological agent, clot lysis experiments were done with supplemental C1INH sufficient to double and triple its physiological concentration. At these higher concentrations, a maximum rate of clot lysis by HisproUK could be induced without causing the fibrinogen degradation, which otherwise accompanied such doses [55]. The C1INH prevention of fibrinogenolysis, but not fibrinolysis, by HisproUK is a unique property for a plasminogen activator.

**Fig. 1:**
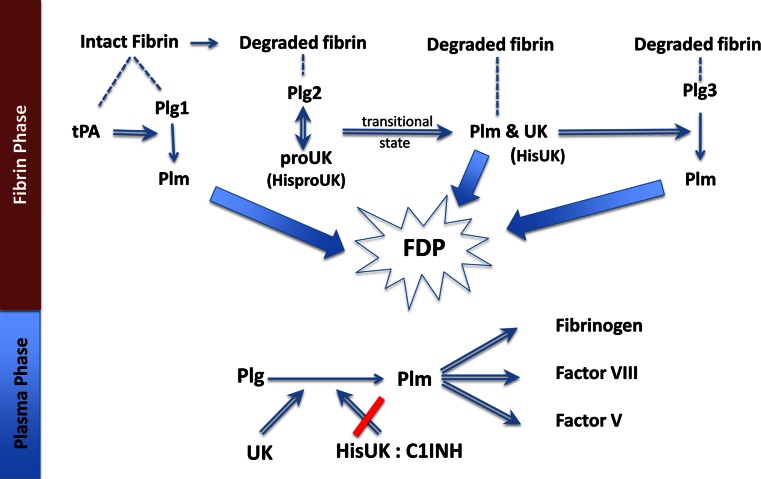
Fibrinolysis by the sequential modes of action of tPA and proUK or HisproUK: tPA binds (*dashed line*) to intact fibrin adjacent to plasminogen(Plg1), forming a ternary complex and activating Plg1 to plasmin (Plm). This initiates fibrin degradation resulting in the creation of new plasminogen binding sites on fibrin. Plg2 binds to a high-affinity binding site on the fibrin E (FFE) domain, which induces a special conformational shape change in Plg2 that enables it to be activated by the intrinsic activity of HisproUK. This HisproUK:plasminogen complex results in Plm formation and the reciprocal activation (*double arrow*) of HisproUK → HisUK, associated with a transitional state that is hypercatalytic. UK, being a non-specific enzyme, then activates the remaining fibrin-bound plasminogen (Plg3). The three Plm together complete fibrin degradation (thick arrows) forming soluble fibrin degradation products (FDP). In the Plasma Phase, when UK diffuses off the fibrin clot, if it is not inhibited, it will activate plasma Plg to Plm, resulting in the degradation of 3 clotting factors, among other side effects. When HisproUK is used in place of proUK, this non-specific effect is prevented due to HisUK being inhibited by C1INH, whose plasma concentration is >1000-fold greater than that of the UK inhibitor

C1INH is a relatively abundant (~250 µg/ml) plasma inhibitor, whereas plasminogen activator inhibitor-1 (PAI-1), the principal inhibitor of UK, is present only at a nanogram concentration (21 ± 7 ng/ml). Since HisproUK is activated to HisUK during clot lysis, it’s inhibition by plasma C1INH helps prevent the bleeding complications that occurred due to UK (see Fig. 1). Since C1INH is also an acute phase reactant, it is apt to be present at higher concentrations in patients with AMI, stroke or thromboembolism, so that supplemental C1INH is less likely to be needed, especially at the lower HisproUK doses needed with the synergistic combination.

### Testing fibrinolytic synergy of tPA and HisproUK

In a clot lysis model in a plasma milieu, the dose of tPA or HisproUK alone which induced the shortest clot lysis time possible was established. Thereafter, the fractional dose combination that gave a comparable lysis time was selected and consisted of 3–6 % of the tPA dose combined with 40 % that of the HisproUK. These fractional doses were almost identical to those of tPA and proUK in the clinical PATENT trial, which were 5 and 40 %, as described above. This similarity between the findings from this in vitro study to those found clinically provided evidence for the clinical relevance of the clot lysis model [56].

In the HisproUK clot lysis study, increasing the mini dose of tPA in the combination had no effect on the lysis time, a finding consistent with tPA’s fibrinolytic function being limited to the initiation of lysis. The doses of tPA and HisproUK in the synergistic combination caused no fibrinogenolysis, in contrast to that which was caused by the doses need of either activator used alone to induce a similar lytic effect [53].

## Conclusions

Intravascular thrombosis remains a leading cause of death and disability for which thrombolysis is the only pharmacological remedy. The thrombolytic, tPA, has become essentially synonymous with thrombolysis but its use, or that of one of its longer half-life derivates, has been declining due to its inadequate efficacy in AMI, incompatibility with PCI, limited efficacy and risk of ICH in ischemic stroke, and too high a bleeding risk for most patients with venous thromboembolism. Instead, intra-arterial devices have become the treatment of choice in AMI are becoming more frequently used in ischemic stroke as well. The resort to these time-consuming methods to treat very time-sensitive conditions is a reflection on the inadequacy of current thrombolysis.

In retrospect, the conclusion that the results of the tPA mega trials “should be taken as evidence that any mortality differences… between different fibrinolytic regimens are unlikely to be large” [16] was a misinterpretation. Instead, the evidence suggests that it is the fibrin-specific mode of action of tPA which restricted its plasminogen activation thereby giving a non-specific activator like SK, with no such restrictions, an efficacy that was essentially comparable but for different reasons. Therefore, the impression of comparability reflected the fibrinolytic limitations of tPA monotherapy.

The biological plasmin-mediated pathway is the basis of all therapeutic thrombolysis and in nature it utilizes both plasminogen activators, tPA and proUK, in a sequential combination rather than only one. Using this paradigm, a synergistic thrombolytic effect was obtained from fractional doses of the activators both in vitro as well as in a clinical study of AMI.

The other activator, proUK, was unstable in plasma at pharmacological concentrations and a more stable single site substitution mutant was designed to replace it. Preclinical testing showed that HisproUK was comparably synergistic as proUK with a much reduced liability for bleeding complications due to a unique property that its enzymatic form, HisUK, was inhibited by C1INH in plasma.

The combination of mini-dose tPA followed by an infusion of a moderate dose of HisproUK is expected to provide more effective therapeutic thrombolysis with a significantly lower risk of ICH, capable of administration without any delay in stroke. Other indications include facilitated PCI, and the treatment of venous thromboembolism.
